# Medical Spiritualism

**Published:** 1857-08

**Authors:** 


*Medical Spiritualism.*—In a late number of the Boston Medical and Surgical Journal, there is a very interesting paper by Dr. Walter Channing, on Medical Spiritualism, in which the writer records a notable instance of the attainments of "medical mediums," one of whose exhibitions he attended.

The medical medium had in consultation three distinguished doctors whom he had called from the spirit sphere. A few questions put to the medium as to what he saw in the organism of the patient, effectually exposed his utter ignorance of anatomy, and the little aid the spirits afforded him in making up the deficiency. After some further conversation, the medium was requested to ask Dr. ——, one of the medical ghosts in consultation, what he thought of the "hour-glass contraction"—a very dangerous condition sometimes induced after child-birth—but not, by its name, suggesting to an unprofessional person any idea of its real character. The medium pondered some time upon the question, in order, apparently, to make sure of the departed doctor's present opinion, and then said: "Dr. —— thinks hour-glass contractions a very good thing, and should certainly recommend it."

